# Pathological complete remission in ALK-positive lung cancer patient after multiple lines of conversion therapy

**DOI:** 10.3389/fonc.2022.967675

**Published:** 2022-11-29

**Authors:** Dan Que, Hongbo Zou, Bijing Mao, Huan Zhang, Wei Liang, Qin Liu, Leiyu Ke, Lijie Guo, Qichao Xie

**Affiliations:** ^1^ Department of Oncology, The Third Affiliated Hospital of Chongqing Medical University, Chongqing, China; ^2^ Medical Department, Shanghai OrigiMed Co., Ltd, Shanghai, China

**Keywords:** multiple lines of therapy, lung adenocarcinoma, pathological complete remission, targeted therapy, ALK+

## Abstract

**Introduction:**

Traditional therapeutic approaches for the treatment of advanced non-small-cell lung cancer (NSCLC) are based on chemotherapy. However, the discovery and understanding of oncogenic driver alterations has led to the development of targeted therapies that have substantially improved patient outcomes. Still, to date, there have been no reports of patients with advanced anaplastic lymphoma kinase (ALK)-positive lung cancer achieving clinical complete response (cCR) in the systemic lesion and pathological complete remission (pCR) in primary lung lesion after multiple lines of conversion therapy.

**Methods:**

In this case, a 55-year-old man was diagnosed with ALK-positive, stage IV lung adenocarcinoma using immunohistochemistry and next generation sequencing (NGS) tests.

**Results:**

Crizotinib and two other ATP-competitive ALK inhibitors, ceritinib and alectinib, were used respectively as first-line, second-line, and third-line therapy. The patient received treatment with crizotinib and achieved partial response (PR), but 5 months later the efficacy was evaluated as progressive disease (PD). Ceritinib was used as the second-line treatment, but the disease progressed 6 months later. Alectinib was used as the third-line treatment, but the efficacy was evaluated as PD. From April 2019 to November 2019, the patient received 4 cycles of induction chemotherapy with pemetrexed/carboplatin/bevacizumab and then switched to pemetrexed/bevacizumab as the fourth-line treatment, and received the fifth line treatment, cetuximab/paclitaxel liposome/nedaplatin, for 1 cycle, but the disease still progressed. Then the patient received the sixth line of treatment, camrelizumab/lorlatinib, for 9 antitumor cycles, resulting in PR. The patient underwent surgery followed by maintenance treatment with lorlatinib and achieved cCR. To our knowledge, this is the first documented case of cCR in a patient with ALK-positive advanced lung adenocarcinoma treated with multiple lines of therapy followed by surgical treatment.

**Discussion:**

This case reveals the possible survival benefit of immunotherapy after multiple line treatment in ALK-positive advanced lung adenocarcinoma, indicating that it is possible find new therapeutic targets based on NGS molecular detection and provide precise therapeutic strategies for clinical practice when drug resistance or progression occurs in cancer therapy.

## Introduction

Lung cancer is one of the most common types of cancer and the leading cause of cancer-related death worldwide, with the most common type being adenocarcinoma, accounting for approximately more than 40% of all lung cancer cases (1, 2). Despite the progress in understanding the pathogenesis of lung adenocarcinoma and the recent advances in the field of targeted therapy that have brought about new therapeutic options and methods for its treatment, lung adenocarcinoma remains one of the high rate of metastasis and invasiveness types, and the prognosis of lung adenocarcinoma continues to be frustrating, and its 5-year survival is less than 20% (1, 3–5).

Over the last decade, innovative targeted therapies acting on oncogenic drivers have significantly improved prognosis compared to systemic chemotherapy (6). Personalized therapy based on molecular markers has completely transformed the therapeutic landscape in advanced non-small-cell lung cancer (NSCLC). However, patients with NSCLC are usually diagnosed at advanced stages, and median survival time after diagnosis is usually less than 1 year. It is now generally recommended that all lung cancer patients eligible for chemotherapy undergo molecular testing to determine the best clinical treatment options. The standard of care consists largely of systemic treatment modalities, such as concomitant chemo- and radiotherapy for the majority of patients in stage III, and targeted therapy, chemotherapy, immunotherapy, or chemo-immunotherapy for stage IV patients and some stage III NSCLC patients (7). However, salvage thoracic surgery may still be a viable option for advanced NSCLC beyond frontline treatment as the disease progresses due to drug resistance.

Anaplastic lymphoma kinase (ALK)-positive NSCLC patients account for approximately 3%−7% of all NSCLC cases (8). Several highly selective ALK-tyrosine kinase inhibitors (ALK-TKI), such as ceritinib, alectinib, and lorlatinib, have clinical activity against several secondary ALK mutations related to drug resistance, such as G1202R, G1269A, and C1156T (9). A comparison of crizotinib treatment with chemotherapy in previously untreated patients and patients with progressive disease during previous chemotherapy demonstrated that crizotinib is more effective than chemotherapy in patients with ALK-rearranged NSCLC (10, 11). A retrospective analysis of the relevance of surgical resection after ALK inhibitor therapy showed a relapse-free survival of 15 months after salvage thoracic surgery (12). There is a lack of evidence to assess the safety and efficacy of surgery in ALK-altered, progressive advanced NSCLC. Here, we describe the multidisciplinary treatment and outcome of a stage IV lung adenocarcinoma patient harboring an ALK alteration. Thoracoscopic-assisted left pneumonectomy, lymph node dissection, and pleural adhesion ligation were performed after sixth-line therapy. At the time of manuscript preparation, the patient had no evidence of recurrence or progression with a progression-free survival of 24 months.

## Patient and sequencing

A 51-year-old man was examined at a local hospital on January 22, 2018 due to a dry cough and fever. Patients were initially diagnosed with single gene detection, ALK-V (D5F3) (+), ROS-1 (–) and PD-L1 (+, 10%) were determined using immunohistochemical (IHC). *ALK* gene was detection by IHC using Ventana-D5F3, and EGFR (–) was determined using amplification refractory mutation system (ARMS) method. On July 23, 2018, secondary gene detection was performed after 5 months of first-line treatment, and mutation analysis of lung cancer 56 gene was performed using Capture-based NGS testing: EML4-ALK (E18: A20) gene fusion, abundance 49.37%; ALK exon 25 p. G1269A missense mutation, abundance 3.62%; and ALK exon 23 p. R1192 missense mutation, abundance 4.10%. The p. R130L missense mutation in PTEN exon 5 was present in 5.06% abundance. No mutations were detected in the remaining genes including TP53. Formalin-fixed paraffin-embedded (FFPE) tumor tissues and matched blood samples were obtained and delivered to OrigiMed (Shanghai, China) for 300+ gene testing and MyGenostics (Beijing, China) for 595 gene panel testing for genetic alteration determination. We found 3.36% abundance of EML4-ALK (E18: A20) gene fusion and3% abundance of EGFR amplification in, but none of them detected *TP53* gene mutation. TMB was calculated by counting somatic mutations including coding base substitutions and indel mutations per megabase (muts/Mb) of genome examined, excluding known hotspot mutations in oncogenic drivers and known germline alterations in the single nucleotide polymorphism database. The study was approved by the Institutional Review Board and carried out in accordance with the principles of the Declaration of Helsinki. Our study was approved by the Ethics Committee of the Third Affiliated Hospital of Chongqing Medical University (Ethics No.: 2022-57-01), Informed consent was obtained from this patient.

Genomic DNA of tumor samples and white blood cells from matched blood was extracted using the QIAamp DNA FFPE Tissue Kit and QIAamp DNA Blood Midi Kit (Qiagen, Hilden, Germany), and the testing was performed in OrigiMed Co., Ltd (Shanghai, China), which was a laboratory certified by the College of American Pathologists and Clinical Laboratory Improvement Amendments.

## Results

**Figure 1:**
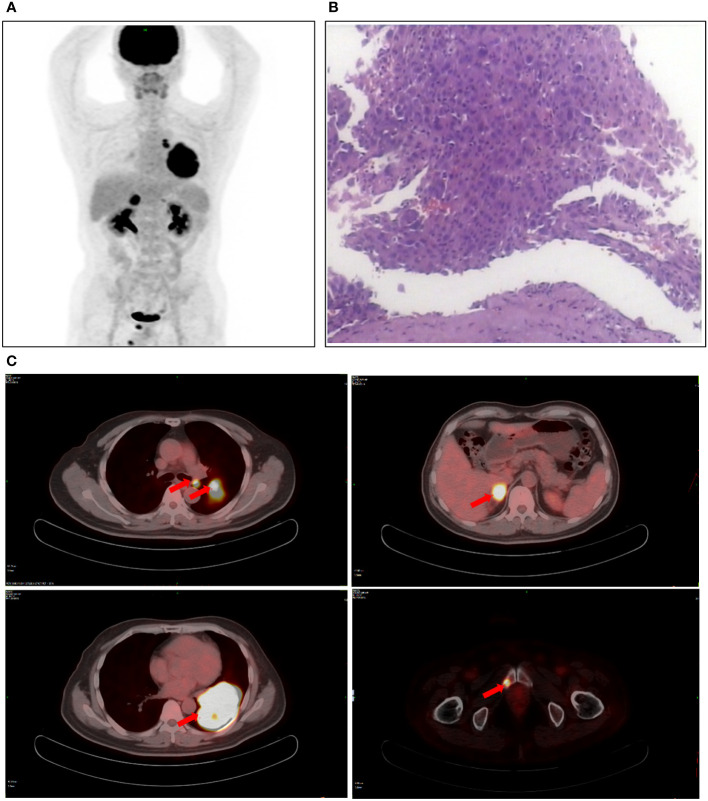
Patient examination before treatment. **(A)** PET-CT scan showed a space-occupying lesion in the right hilum with obstructive pneumonia. **(B)** The lesions were considered lung adenocarcinoma based on fiberoptic biopsy and HE staining. **(C)** The PET-CT observed a soft mass in the left lower lobe, a soft tissue density nodule in the right adrenal gland, and increased local metabolism in the right superior pubic branch.

**Figure 2:**
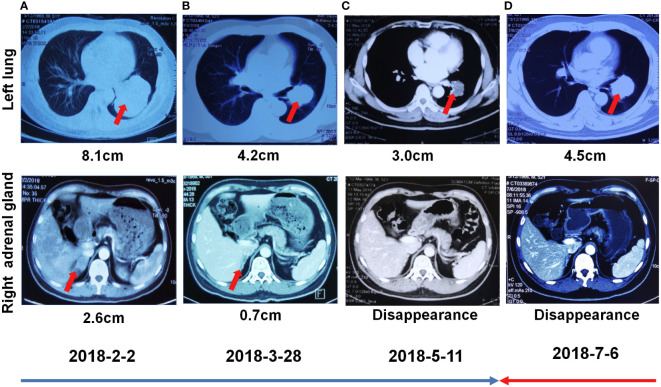
First-line treatment with crizotinib. **(A)** Chest CT showed focal changes in the left lung and right kidney on February 02, 2018. **(B)** Chest CT showed focal changes in the left lung and right kidney on March 28, 2018. **(C)** Chest CT showed focal changes in the left lung and right adrenal gland on May 11, 2018. **(D)** Chest CT showed focal changes in the left lung and right kidney on July 6, 2018.

**Figure 3:**
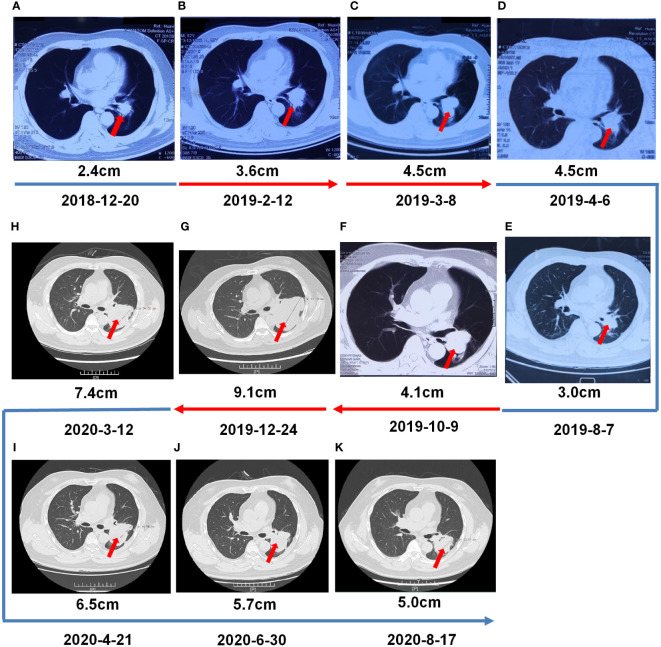
Multiple-Line Systemic Treatment. **(A, B)** Second -line treatment with ceritinib. **(C)** The third- line treatment with alectinib. **(D–F)** The fourth-line systemic treatment from 4 cycles of induction chemotherapy (pemetrexed/carboplatin/bevacizumab), and then switched tpemetrexed/bevacizumab. **(G)** Fifth-line treatment with cetuximab/paclitaxel liposome/nedaplatin. **(H–K)** Sixth-line treatment with camrelizumab/lorlatinib.

**Figure 4:**
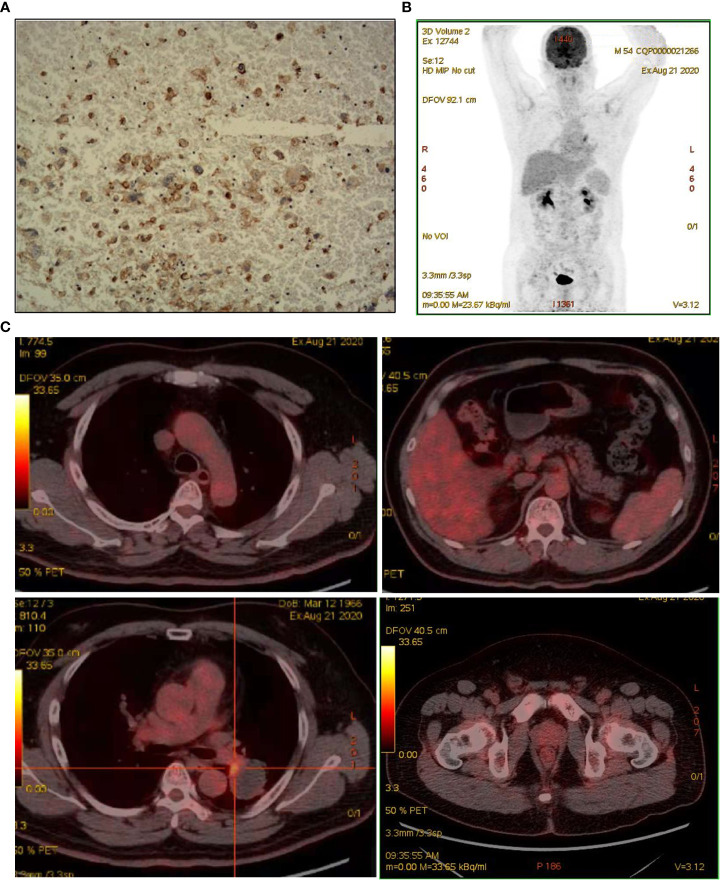
Assessed after multiple lines of treatment. **(A)** IHC for PD-L1 staining. **(B, C)** Repeat PET/CT revealed patchy increased radioactive uptake in the hilar aspect of the left lung mass and radioactive uptake defect area in the central area of the mass.

**Figure 5:**
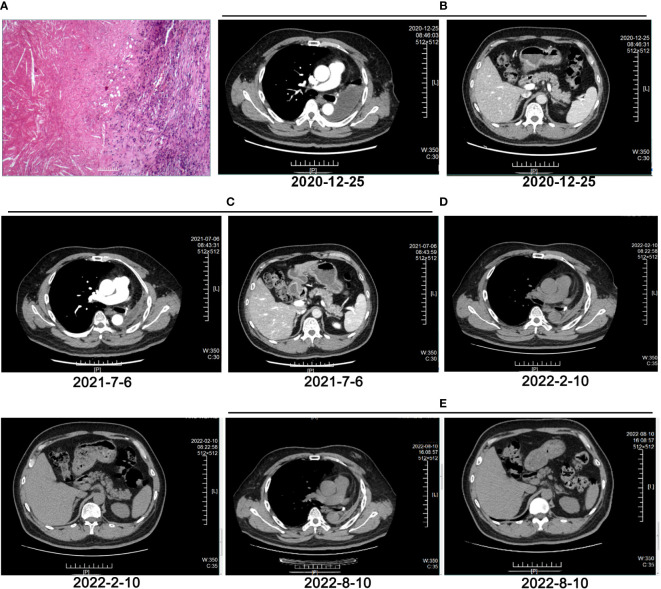
Evaluation after surgery and adjuvant therapy. **(A)** HE staining of pathological tissues after multiple lines of treatment and surgery. **(B)** CT image of left lung on December 25, 2020. **(C)** CT image of left lung on July 6, 2021. **(D)** CT image of left lung on February 10, 2022. **(E)** CT image of left lung on August 10, 2022.

**Figure 6:**
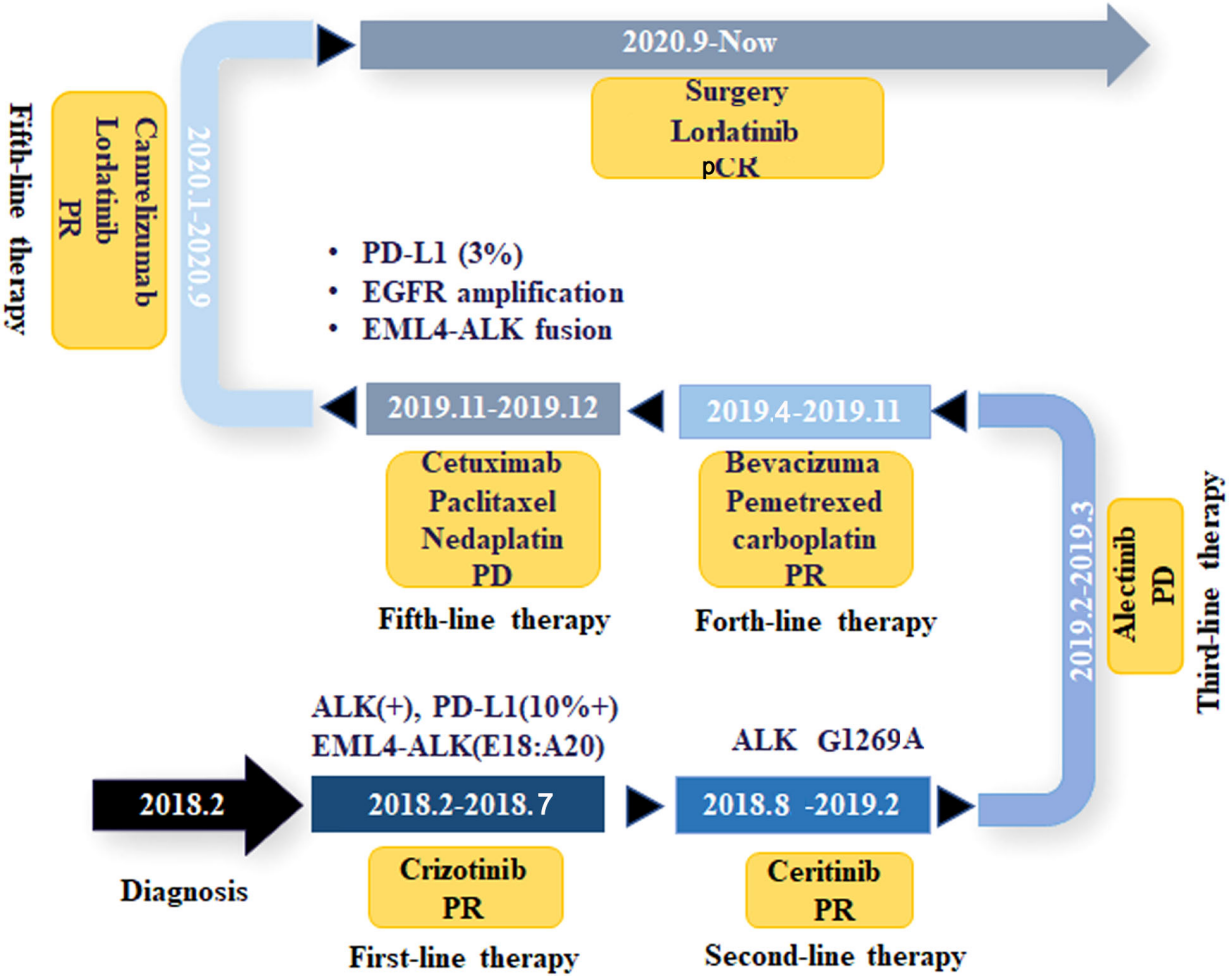
The timeline with therapy and disease status. PD, progressive disease; PR, partial response; pCR, pathological complete remission.

A 55-year-old man, a non-smoker, who described recurrent dry coughing over a year and fever for one day was examined at a local hospital on January 22, 2018. After fourth-line therapy, he was referred to our hospital. This patient weighed 62 kg and his PS score was 0. His PET-CT scan showed a space-occupying lesion in the right hilum with obstructive pneumonia (**Figure 1A**). A PET-CT showed a soft mass in the left lower lobe with increased glucose metabolism (5.5 × 7.7 cm), and the parapulmonary trunk and left hilar lymph nodes showed increased glucose metabolism (maximum diameter 0.7 cm), a soft tissue density nodule in the right adrenal gland (2.1 × 2.6 cm) showed increased glucose metabolism, and increased local metabolism in the right superior pubic branch (1.0 cm) (**Figure 1C**). The lesions were considered lung adenocarcinoma based on fiberoptic biopsy and hematoxylin-eosin staining (**Figure 1B**). Molecular testing results showed that *ALK and* PD-L1 were positive, and *ROS-1* and *EGFR* were negative. Finally, the patient was diagnosed with stage IV *ALK*-positive advanced lung adenocarcinoma with metastatic malignancy in the right adrenal gland. He was treated with crizotinib (250 mg BID) on February 14, 2018. The mass in the left lower lobe was reduced from 8.1 cm to 4.2 cm, and the right adrenal tumor was reduced from 2.6 cm to 0.7 cm on March 28, 2018, achieving partial response (PR) according to the Response Evaluation Criteria In Solid Tumors (RECIST) 1.1 criteria (**Figures 2A, B**). On May 11, 2018, the mass in the left lower lobe shrank to 3.0 cm, while the right adrenal metastases disappeared with no recurrence up to follow-up time, and the response evaluation was still PR (**Figure 2C**). But the left lower lobe mass increased to 4.5 cm, and the efficacy qualified as progressive disease (PD) after 5 months (**Figure 2D**). The main side effects were vomiting and diarrhea, and disappeared after 2 months. Molecular testing showed an *EML4-ALK* (E18:A20) fusion, and PD-L1 positivity, with a tumor proportion score (TPS) of 10% on IHC. In view of the accessibility of drugs, medical insurance reporting and other factors, ceritinib was deemed the ideal treatment. From August 2018 to February 2019, the patient received the second-line treatment of ceritinib (450 mg PO QD), without significant adverse reactions during medication, and progressed after 6 months. On December 20, 2018, it continued to decrease to 2.4 cm, and was deemed PR. However, on February 12, 2019, the lesion in the left lower lung increased to 3.6 cm, and the disease progressed (**Figures 3A, B**). From February 2019 to March 2019, alectinib (600 mg PO BID) was used as the third line therapy, while the efficacy was evaluated as PD (**Figure 3C**). The NGS analysis revealed an *EML4* exon20-*ALK* exon20 fusion. From April 2019 to November 2019, the patient received the fourth-line treatment, including 4 cycles of induction chemotherapy (pemetrexed 835 mg/carboplatin 500 mg/bevacizumab 485 mg), and maintenance therapy (pemetrexed 835 mg/bevacizumab 485 mg). After induction chemotherapy, the patient felt fatigue and anorexia. On August 7, 2019, the lesion in the left lower lung shrank was reduced to3.0 cm and evaluated as PR. However, the left lower lung lesion was enlarged (4.1 cm), and the response was evaluated as PD on October 9, 2019 (**Figures 3D–F**). Molecular testing showed *EGFR* amplification and *-EML4*-*ALK* fusion, the TMB was 1.6 muts/Mb and the tumor was PD-L1 positivewith a TPS of 3% on immunohistochemistry (**Figure 4A**). The patient received the fifth line of treatment (cetuximab 700 mg/paclitaxel liposome 210 mg/nedaplatin 100 mg) for 1 cycle. After treatment, the patient had rash, fatigue, and anorexia. After 1 month, the left lower lung lesion enlarged to 9.1 cm (**Figure 3G**). Then the patient received the sixth line of treatment (camrelizumab 200 mg/lorlatinib 100 mg QD) for 9 antitumor cycles, resulting in PR and gaining 15 kg in weight. Side effects included pain in both lower limbs, an NRS pain score of 6, with the disappearance of the above symptoms after 6 months (**Figures 3H–K**). The PET-CT on August 21, 2020 showed patchy, increased radioactive uptake in the left lung mass and a radioactive uptake defect area in the central area of the mass. The left hilar lymph node, right adrenal gland, and right suprapubic branch showed no mass or radioactive uptake (**Figures 4B, C**). After consultation with experts from thoracic surgery, interventional medicine, and the radiotherapy center, surgery was recommended. On September 23, 2020, thoracoscopic-assisted left pneumonectomy, lymph node dissection, and pleural adhesion ligation were performed and postoperative pathological analysis revealed massive necrosis of nodules in the upper and lower lobes of the left lung, no obvious cancer tissue, focal granulomatous inflammation, no cancer at the bronchial resection margin, and no metastasis of the lymph nodes. The pCR was achieved in the primary lung lesion (**Figure 5A**). From October 2020 to now, the patient continues to receive maintenance treatment with lorlatinib (100 mg QD) with good tolerance, no side effects, and an Eastern Cooperative Oncology Group (ECOG) score of 1. The results of chest and abdomen CT and abdominal color Doppler ultrasound monitoring showed no new lesions. (**Figures 5B–E**). During follow-up, this patient remains in cCR in the systemic lesion and a pCR in the primary lung lesion. The timeline of treatments is shown in **Figure 6**.

## Discussion

NSCLC makes up nearly 85% of lung cancer cases and is one of the most lethal forms of malignancies. Surgery is currently the principal NSCLC treatment because it provides a possible cure. Unfortunately, more than 70% of patients are diagnosed with advanced NSCLC (stage III and IV) at diagnosis (13). The advent of multiple molecular targets in advanced NSCLC has brought about new treatments, but no cases of cCR in advanced *ALK*-positive lung cancer after multiple lines of conversion therapy have been reported. In our case, a patient with stage IV lung adenocarcinoma achieved operable evaluation after multiple lines of treatment, and reached cCR after adjuvant therapy after surgery.

As the most common fusion type of *ALK*, *EML4*-*ALK* is responsible for the growth and survival of lung cancer cell lines and functions as a therapeutic target in NSCLC (14). The identification of *EML4*-*ALK* as an oncogenic driver in NSCLC in the clinical development of crizotinib and the observation of promising clinical responses in patients with NSCLC harboring *ALK* translocations accelerated its clinical development in *ALK*-positive NSCLC (15). Crizotinib is the first *ALK*-targeted therapy approved by the US Food and Drug Administration (FDA) for patients with *ALK*-rearranged NSCLC and is also a standard first-line treatment for advanced *ALK*-positive NSCLC in many countries, including China (10, 15). The analysis of the PROFILE 1014 study provides a new benchmark for overall survival (OS) in patients with *ALK*-rearranged NSCLC and highlights the benefit of crizotinib for prolonging survival in this patient population (10). In our case, the patient was treated with crizotinib and achieved PR by RECIST 1.1 criteria. But 5 months later, the efficacy was evaluated as PD. To overcome crizotinib resistance, several next-generation *ALK* inhibitors have been developed, including ceritinib and alectinib, which were developed for patients with *ALK*-positive NSCLC resistant to crizotinib and have been used in China (16). Ceritinib showed significant antitumor and intracranial activity in patients with *ALK*-rearranged NSCLC, including patients who had previously received crizotinib and also received chemotherapy (17). The efficacy of ceritinib was confirmed in patients with *ALK*-rearranged lung cancer, including patients with brain metastases who progressed on chemotherapy and crizotinib (18). In addition to efficacy in patients with *ALK* mutations resistant to crizotinib, ceritinib has also been effective in patients with *ALK*-independent mechanisms of resistance to crizotinib (19). Alectinib showed impressive progression-free survival (PFS) results and is recommended in *ALK*-positive advanced NSCLC patients (20, 21). Alectinib and ceritinib demonstrated significant survival benefits for previously untreated advanced *ALK*-rearranged NSCLC patients (9) Ceritinib and alectinib were approved for use as the second-line and third-line therapy in this patient, but the disease still progressed. A single-arm phase II study of pemetrexed/carboplatin/bevacizumab followed by pemetrexed/bevacizumab maintenance demonstrated efficacy (OS:14.1 months; PFS: 7.8 months) and acceptable safety (22). From April 2019 to November 2019, the patient received 4 cycles of induction chemotherapy with pemetrexed/carboplatin/bevacizumab, and then switched to pemetrexed/bevacizumab as the fourth-line treatment, and received the fifth line of treatment (cetuximab/paclitaxel liposome/nedaplatin) for 1 cycle, but the disease progressed.

Lorlatinib is a potent, brain-penetrant, third-generation inhibitor of *ALK* and ROS1 tyrosine kinases with broad coverage of *ALK* mutations (23). As the first PD-1 antibody approved for lung cancer in China, camrelizumab has proved effective for NSCLC patients (24). Previous studies have reported that ALK gene mutation may induce PD-L1 expression, but ALK gene mutation may have a negative effect on immunotherapy, so patients with ALK mutation were excluded from multiple immunotherapy series such as KEYNOTE, Checkmate, and Impower (25–27). There are also relevant studies of targeted therapy combined with immunotherapy. For example, the combination of alectinib and atezolizumab is feasible, but increased toxicity was found compared with the individual agents (NCT02013219) (28). The cohort study of nivolumab plus crizotinib did not meet the primary endpoint of safety and tolerability for the first-line treatment of ALK (due to the severe hepatic toxicities), but 38% patients had a partial response (26). Erlotinib plus ipilimumab caused excessive short-term gastrointestinal toxicity leading to early study closure. Median OS has not been reached but will be at least 47.2 months from the initiation of crizotinib; therefore, targeted therapies with immunotherapy in NSCLC merit further study (29). There are also several cases demonstrating that patients respond to combined immunotherapy after multiple lines of targeted therapy (30, 31). Afterwards, the patient received the sixth line of treatment (camrelizumab/lorlatinib) for 9 antitumor cycles, resulting in PR, and no significant immune-related adverse reactions were found. It was then recommended to perform surgery, followed by a maintenance treatment with lorlatinib. The patient then achieved a cCR in the systemic lesion and a pCR in the primary lung lesion. To our knowledge, this is the first documented case of cCR in a patient with *ALK*-positive advanced lung adenocarcinoma treated with multiple lines of therapy followed by surgical treatment. Therefore, combined immunotherapy can be tried for patients with ALK mutation who have no better choice after multiple lines of therapy.

In conclusion, the patient with stage IVb lung adenocarcinoma that was treated with multiple therapy lines, including target, immunotherapy, and chemotherapy, and then underwent surgery achieved cCR. This case reveals the possible survival benefit of immunotherapy after multiple lines treatment in *ALK*-positive advanced lung adenocarcinoma, suggesting it is possible to find new therapeutic targets based on NGS molecular detection and provide precise therapeutic strategies for clinical practice when drug resistance or progression occurs in cancer therapy.

## Data availability statement

The data supporting the conclusions of this article will be made available by the authors, without undue reservation.

## Ethics statement

This study has been approved by Ethic Committee of the Third Affiliated Hospital of Chongqing Medical University. The patients/participants provided their written informed consent to participate in this study. Written informed consent was obtained from the individual(s) for the publication of any potentially identifiable images or data included in this article.

## Author contributions

DQ, QCX, HBZ, BJM, HZ, WL and QL all participated in the management of this case. DQ, QCX, LYK and LJG were in charge of manuscript drafting and data collection. DQ and QCX did the modification. All authors contributed to the article and approved the submitted version.

## Conflict of interest

LYK and LJG are employees of OrigiMed.

The remaining authors declare that the research was conducted in the absence of any commercial or financial relationships that could be constructed as a potential conflict of interest.

## Publisher’s note

All claims expressed in this article are solely those of the authors and do not necessarily represent those of their affiliated organizations, or those of the publisher, the editors and the reviewers. Any product that may be evaluated in this article, or claim that may be made by its manufacturer, is not guaranteed or endorsed by the publisher.
